# CARDIO-CEREBROVASCULAR DISEASE RISK FACTORS AMONG MALE NIGHT-SHIFT WORKERS IN SOUTH KOREA: A COMPARATIVE ANALYSIS OF DRIVERS AND SECURITY GUARDS

**DOI:** 10.13075/ijomeh.1896.02453

**Published:** 2025

**Authors:** Jung-Min Sung, Young Joong Kang, Shinhee Ye, Eun-A Kim

**Affiliations:** 1 Korea Occupational Safety and Health Agency, Occupational Safety and Health Research Institute, Ulsan, Republic of Korea; 2 Korea Workers' Compensation and Welfare Service, Department of Occupation and Environment Medicine, K-COMWEL Incheon Hospital, Incheon, Republic of Korea; 3 Korea Medical Institute (KMI), Gangnam Health Checkup Center, Seoul, Republic of Korea; 4 Korea Workers' Compensation and Welfare Service, Department of Occupation and Environment Medicine, K-COMWEL Changwon Hospital, Changwon, Republic of Korea

**Keywords:** metabolic syndrome, cardiovascular diseases, old age, data collection, shift work schedule, environmental and public health

## Abstract

**Objectives:**

This study investigates the prevalence of cardio-cerebrovascular disease (CCVD) risk factors among male night-shift workers in South Korea, focusing on drivers and security guards, who constitute a significant portion of the older worker population.

**Material and Methods:**

Using data from the 2016 nationwide workers' special health examination, the authors analyzed health habits, past illnesses, and body measurements related to CCVDs among male drivers (N = 8862) and security guards (N = 7156) in their 60s.

**Results:**

The age of the drivers and security guards were mean ± standard deviation 63.19±2.62 years and 64.93±2.72 years, respectively. The drivers exhibit unhealthier lifestyle habits and a higher prevalence of diabetes, dyslipidemia, and obesity compared to security guards. Additionally, drivers have unhealthier body measurement values and a higher prevalence of metabolic syndrome (OR = 1.844, 95% CI: 1.722–1.974, p < 0.001).

**Conclusions:**

These findings underscore the heightened risk of CCVD among drivers compared to security guards among older night-shift workers in South Korea, highlighting the need for tailored health policies for this demographic.

## INTRODUCTION

While the global population is aging, South Korea is one of the most rapidly aging societies worldwide. Korea is expected to become a super-aged society, defined as ≥20% of the population aged ≥65 years, by 2025 [1]. Korea has a relatively unstable old-age pension and social security system compared with other developed countries, which has led to the claim that the working status of the older population is highly associated with their health [2]. With the rapidly aging Korean population, increasing numbers of older workers will enter the labor market, and appropriate attention and implementation of measures in response to this aging workforce is necessary. Given the demographic shift towards an aging population, there is a pressing need to focus on and implement measures to address the needs of older workers entering the labor market.

Night-shift work poses several risks to the health and well-being of workers because of disruption to the body's natural circadian rhythms and is associated with various health risks, including cardiovascular effects which may involve complex interactions with dyslipidemia [3], abnormal eating and related obesity [4], sleep disorders [5], and occupational injuries [6]. A recent umbrella review also found highly suggestive evidence of associations between shift work and both myocardial infarction and diabetes mellitus incidence [7]. The International Agency for Research on Cancer (IARC) classifies shift work as a possible human carcinogen [8]. In response to this challenge, the Korean Ministry of Employment and Labor introduced legislation in June 2013 mandating special health examinations for night-shift workers in enterprises with ≥300 employees by 2014, later expanding coverage to enterprises with <50 employees nationwide by 2016. Analyses conducted by Kim et al. [9] highlighted a significant increase in the proportion of older workers, particularly in sectors such as healthcare, business facility management, and transportation, following the implementation of workers' special health examinations (WSHEs) for night-shift workers. The WSHE data store diverse data depending on the type of exposed harmful factor and evaluated target organ. With shift work newly designated as a harmful factor subject to WSHE in South Korea, this change has incorporated substantial data on older male workers, particularly those at risk for cerebrovascular and cardiovascular diseases.

Security and driving occupations represent the largest proportion of night-shift jobs for males. These occupations share common characteristics, including a higher mean age than other professions and present challenges in terms of managing health issues during solitary night shifts. Given the potential ramifications of health risks in the transportation and security sectors leading to serious accidents that directly impact public safety, understanding and managing the health conditions of workers in these occupations are of paramount importance.

As the WSHE includes shift work as a hazardous factor, its role in cardio-cerebrovascular disease (CCVD) has emerged. Therefore, it is necessary to assess the actual CCVD risk among shift workers who undergo the WSHE. This is particularly important in South Korea, where the workforce is aging, and attention should be focused on older workers who are becoming a key issue. The authors investigated the prevalence of CCVD risk factors among male security guards and drivers in an older worker population engaged in night-shift work in Korea. Using data from special health examinations, the authors sought to assess participant health status and underscore the necessity for tailored interventions to safeguard the well-being of this demographic sector.

## MATERIAL AND METHODS

### The health management framework for shift workers and workers exposed to hazardous substances in South Korea

In South Korea, under the Occupational Safety and Health Act, employers are required to conduct workers' general health examinations (WGHE) for their employees: biennially for office workers and annually for non-office workers. In addition to the WGHE, regardless of industry, employers must conduct WSHE for employees exposed to harmful agents that necessitate additional health monitoring. The WGHE include clinical examinations and surveys (e.g., past medical history, smoking and alcohol consumption, exercise habits), physical measurements (height, weight, waist circumference), blood pressure, vision, hearing, chest X-rays, blood tests (fasting blood glucose concentration, hemoglobin, aspartate transaminase [AST], alanine aminotransferase [ALT], γ-glutamyltransferase [γ-GTP], serum creatinine, estimated glomerular filtration rate [e-GFR]), and urine protein tests.

The conventional WSHE aims to detect diseases and symptoms early by conducting medical tests for physical symptoms affected by harmful agents, clinical examinations of target organs, surveys, related medical tests, and monitoring biological exposure indicators. Key hazardous factors include noise, asbestos and other harmful dust, as well as organic compounds, heavy metals, acids, alkalis, and electromagnetic and radiofrequency waves associated with occupational cancers and diseases. The purpose of WSHE is to evaluate job suitability and protect workers by maintaining exposure levels below permissible limits.

In 2014, night-shift work was legally designated as a hazardous occupational environment requiring special health examinations in South Korea [10]. Newly included among the harmful factors subject to special health examinations is shift work, defined as follows:

– performing 8-hour shifts, including work between midnight and 5 a.m., at least 4 times/month on average over 6 months,
– working between 10 p.m. and 6 a.m. for an average of ≥60 h/month.

The target organs for shift work include the nervous system (sleep disorders), gastrointestinal system, cardiovascular system, and endocrine system (e.g., female breast cancer). The aim is not to strongly restrict shift work but rather to detect diseases early in shift workers and manage their overall health, including underlying conditions. For shift workers, the WSHE involves clinical evaluations, surveys for early detection of sleep disorders, gastrointestinal diseases, and breast cancer, as well as measurements for blood pressure, fasting blood glucose, and cholesterol concentration related to cerebrovascular and cardiovascular diseases. If health examination items overlap between general and special health examinations, they can be substituted within the matching health examination cycle. The WSHE should be conducted at authorized medical institution. These medical institutions are legally required to report the results to a server system managed by an affiliated agency, the Occupational Safety and Health Research Institute (OSHRI) under the Ministry of Employment and Labor. The data transmitted by the medical institution includes the minimum necessary personal information, Job Information Based on the fifth Korean Standard Classification of Occupations by Statistics Korea (KSCO) classification [11] recorded by the medical institutions following WSHE interview, and the WSHE results related to hazardous factors.

### Study population and materials

**Figure 1.**
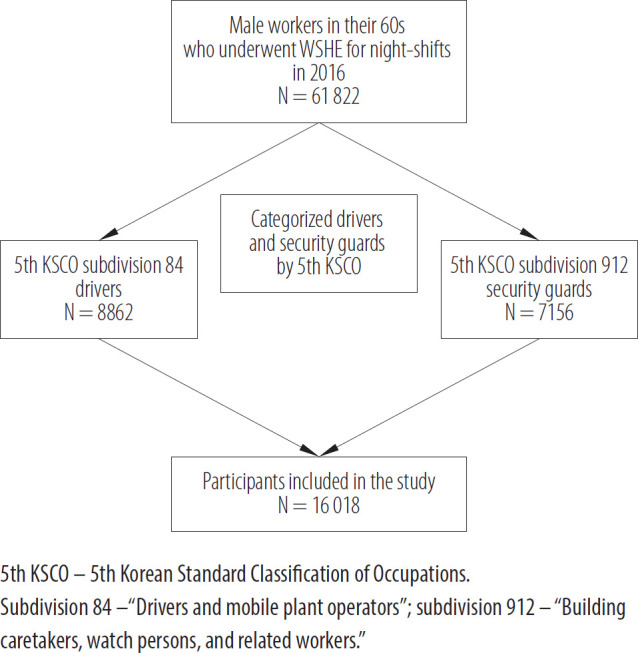
Participant selection of male night-shift workers in their 60s who underwent the Workers' Special Health Examination (WSHE) in South Korea, 2016

For this study, the authors used data reported by OSHRI in 2016. A total of 61 822 male workers in their 60s underwent WSHE for night-shift work. The authors categorized 8862 drivers and 7156 security guards according to the fifth Korean Standard Classification of Occupations by Statistics Korea [11]. The driver and security guard jobs were assigned to subdivisions 84 and 912, respectively (Figure 1). The authors compared WSHE results to analyze the following risk factors for CCVD among driver and security guard respondents:

– duration of night-shift work,
– alcohol consumption,
– smoking habits,
– past illness,
– exercise habits,
– waist circumference,
– obesity with a body mass index (BMI) of ≥25 kg/m^2^ (globally, WHO defines obesity as a BMI ≥30 kg/m^2^, however, for Asian populations, including South Koreans, lower BMI cut-off points are recommended due to higher body fat percentages at the same BMI levels compared to Western populations – in South Korea, obesity is defined as a BMI ≥25 kg/m^2^)[12],
– blood pressure,
– glucose concentration,
– cholesterol concentration.

These factors were assessed through the respondents' answers during the WSHE, and measurements were conducted by medical staff. Low-density lipoprotein (LDL) cholesterol concentrations were calculated using the Friedewald equation [13] as follows:


(1) $$\begin{array}{c} \text{LDL}\;\text{cholesterol}=\text{total}\;\text{cholesterol}-\\ \text{HDL}\;\text{cholesterol}-(\text{TG}/\text{S}) \end{array}$$

where:

HDL – high-density lipoprotein,
TG – triglyceride.

The duration of night-shift work was classified as <5 years, 5–10 years, or >10 years. A high-risk drinking habit (binge drinking) was defined as twice a week or more, with a consumption of >7 glasses (50 ml of Korean soju or 200 ml of beer) on each occasion. Physical activity habits were broadly classified as vigorous, moderate, or aerobic. Vigorous-intensity physical activities included heavy breath-taking activities, such as running, biking at high speed, and mountain climbing, lasting for ≥20 min. Moderate-intensity physical activities were defined as breath-taking activities, such as fast walking, biking at moderate speed, and cleaning the floor on hands and knees for ≥30 min. Aerobic activities were defined as light exercises such as walking. Respondents who engaged in moderate- to vigorous-intensity physical activities were classified into the physical activity group. Respondents who conducted ≥3 sessions of vigorous-intensity activity or ≥5 sessions of moderate-intensity physical activity were included. For the measurement of blood pressure, fasting blood glucose, and blood cholesterol, hypertension was defined as high systolic blood pressure (≥140 mm Hg) or high diastolic blood pressure (≥90 mm Hg). Type 2 diabetes was defined as a fasting blood glucose concentration ≥126 mg/dl. Dyslipidemia was defined as total cholesterol concentration ≥240 mg/dl. Current medication use for hypertension, diabetes, and dyslipidemia was also recorded in this study.

The criteria for metabolic syndrome (MetS) followed those of the International Diabetes Federation (IDF) [14]. According to the IDF definition for MetS, the presence of ≥3 of the following risk determinants should be considered:

– increased waist circumference ≥90 cm for men, ≥80 cm for women),
– elevated TG concentrations (≥150 mg/dl) or specific treatment for this lipid abnormality,
– reduced HDL cholesterol (men <40 mg/dl, women <50 mg/dl) or specific treatment for this lipid abnormality,
– increased blood pressure (≥130/≥85 mm Hg) or previously diagnosed hypertension,
– raised fasting plasma blood glucose (≥100 mg/dl) or previously diagnosed type 2 diabetes.

While verifying the respondents' cholesterol regulation, it was not possible to ascertain whether participants were being treated for TG, LDL, or HDL concentrations. Therefore, respondents who reported taking cholesterol-regulating medications could fall into category 2, category 3, or they could fall into both categories 2 and 3. If the respondent only took medication for elevated LDL cholesterol, neither category 2 nor category 3 applied. In cases where participants within the normal range for blood cholesterol reported taking medications for dyslipidemia, it was defined as applicable to only category 3 with reduced HDL cholesterol determinants to avoid redundancy.

### Statistical analyses

The authors compared the WSHE results of male nightshift drivers and security guards in their 60s. Continuous variables are presented as means (M) ± standard deviations (SD), and Student's t-test was employed to assess differences between variables. Categorical variables were analyzed using the χ^2^ test. Odds ratio analysis of the prevalence of MetS in both groups was performed using multiple logistic regression.

Statistical analyses were performed using SAS 9.4 for Windows statistical software (SAS Institute, Cary, NC, USA). Statistical significance set at p < 0.05 was adopted throughout the study.

## RESULTS

### General characteristics of male night-shift workers in their 60s

Table 1 presents the mean age and classification of night-shift work duration for drivers and security guards. The mean age gap between the 2 groups was <2 years. Drivers' age was M±SD 63.19±2.62 years, whereas security guards had a mean age of 64.93±2.72 years (p < 0.01). Notably, 55.34% of drivers and 85.06% of security guards had worked for <5 years. The duration of night shifts for male security guards in their 60s was considerably shorter than that of male night-shift drivers (p < 0.01).

**Table 1.** General characteristics of male night-shift workers in their 60s who underwent the Workers' Special Health Examination (WSHE) in South Korea, 2016

Variable	Participants (N = 16 018)		p
	drivers (N = 8862)	security guards (N = 7156)	
Demographic			
age [years] (M±SD)	63.19±2.62	64.93±2.72	<0.001
work duration [n (%)]			<0.001
<5 years	4904 (55.34)	6087 (85.06)	
5–10 years	1746 (19.7)	681 (9.52)	
>10 years	2204 (24.87)	250 (3.49)	
not answered	8 (5.48)	138 (1.93)	
Health behavior			
binge drinking [n (%)]	1460 (16.47)	955 (13.35)	<0.001
smoking [n (%)]			<0.001
current smoker	4198 (47.37)	1767 (24.69)	
ex-smoker	2760 (31.14)	3164 (44.21)	
non-smoker	1904 (21.48)	2225 (31.09)	
physical activity			
regular [n (%)]	3346 (37.76)	3312 (46.28)	<0.001
intensity [days/week] (M±SD)			
high	2.25±1.77	2.42±1.84	<0.001
moderate	2.36±1.80	2.65±1.92	<0.001
low	3.31±2.17	4.55±2.44	<0.001
Medical			
hypertension [n (%)]	3584 (40.44)	3251 (45.43)	<0.001
diabetes [n (%)]	2829 (31.92)	1598 (22.33)	<0.001
dyslipidemia [n (%)]	1532 (17.29)	834 (11.65)	<0.001
obesity [n (%)]	3992 (45.05)	2638 (36.86)	<0.001

### Comparison of risk behaviors for cardiovascular diseases

A comparison of the risk factors affecting CCVD among night-shift drivers and security guards in their 60s revealed that security guards had healthier lifestyle habits than drivers. Drivers exhibited a higher prevalence of high-risk drinking than security guards (p < 0.01). Additionally, drivers had higher rates of current smoking (47.37%) than security guards (24.69%). Security guards reported engaging in more vigorous, moderate, and aerobic physical activities, with 46.28% of guards regularly performing moderate-to-vigorous-intensity activities compared to 37.76% of drivers (p < 0.01).

### Incidence of underlying conditions in CCVD

Table 1 shows the incidence of chronic CCVD-related diseases among male night-shift workers in their 60s. The incidence of high blood pressure was significantly higher among security guards (45.43%) than that in drivers (40.44%) (p < 0.01). Conversely, drivers had a significantly higher incidence of diabetes, dyslipidemia, and obesity with a BMI ≥ 25 kg/m^2^. Most risk factors, except for hypertension, were more frequent in the driver group.

### Body measurement values and laboratory test data related to CCVDs

Table 2 displays mean values of body measurements and diagnostic tests related to CCVDs, indicating that security guards generally had healthier indicators than drivers. However, security guards had a higher mean systolic blood pressure than drivers (p < 0.01). Nonetheless, security guards showed significantly better body measurement values and overall laboratory data, including mean waist circumference, BMI, and concentrations of total cholesterol, TG, HDL cholesterol, LDL cholesterol, and fasting blood glucose.

**Table 2.** Mean values of body measurements and laboratory tests related to cardiovascular and metabolic health of male night-shift workers in their 60s who underwent the Workers' Special Health Examination (WSHE) in South Korea, 2016

Variable	Participants (N = 16 018)		p
	drivers (N = 8862)	security guards (N = 7156)	
Waist circumference [cm] (M±SD)	87.92±12.79	84.84±7.46	<0.001
BMI [kg/m^2^] (M±SD)	24.69±3.20	24.16±2.84	<0.001
Blood pressure [mm Hg] (M±SD)			
systolic	124.1±12.69	127.7±13.54	<0.001
diastolic	77.21±7.86	78.20±9.03	<0.001
Triglycerides [mg/dl] (M±SD)	177.4±115.5	139.4±94.93	<0.001
Cholesterol [mg/dl] (M±SD)			
total	193.0±41.86	186.9±37.14	<0.001
HDL	49.66±13.16	54.17±16.44	<0.001
LDL	107.9±39.86	104.9±36.64	<0.001
Fasting blood glucose [mg/dl] (M±SD)	118.4±50.06	109.6±30.72	<0.001

HDL – high-density lipoprotein; LDL – low-density lipoprotein.

### Comparison of MetS and underlying diseases in CCVD

Table 3 presents the prevalence of MetS and its components in both groups. The rate of MetS among drivers was significantly higher than that among security guards (p < 0.01). Drivers had a significantly higher rate of truncal obesity (p < 0.01), decreased concentrations of HDL cholesterol (p < 0.01), and elevated TG concentrations (p < 0.01) than security guards.

**Table 3.** Categorical risk factors including components of metabolic syndrome of male night-shift workers in their 60s who underwent the Workers' Special Health Examination (WSHE) in South Korea, 2016

Metabolic syndrome	Participants (N = 16018) [n (%)]		p
	drivers (N = 8862)	security guards (N = 7156)	
Total	4204 (47.44)	2369 (33.11)	<0.001
Component			
truncal obesity^a^	3640 (41.07)	1820 (25.43)	<0.001
elevated blood pressure^b^	5540 (62.51)	4757 (66.48)	<0.001
elevated blood glucose^c^	5655 (63.92)	4255 (59.46)	<0.001
low HDL-cholesterol^d^	2068 (23.34)	1202 (16.8)	<0.001
elevated triglycerides^e^	4379 (49.41)	2353 (32.88)	<0.001

HDL – high-density lipoprotein.

a Waist circumference ≥ 90 cm.

b Systolic blood pressure ≥130 mm Hg/diastolic pressure ≥85 mm Hg or previously diagnosed hypertension.

c Fasting plasma blood glucose ≥100 mg/dl or previously diagnosed type 2 diabetes.

d HDL cholesterol <40 mg/dl or specific treatment for lipid abnormality.

e TG ≥150 mg/dl.

### Prevalence comparison of MetS between night-shift security guards and drivers in their 60s

Table 4 shows the relationship between the 2 groups and prevalence of MetS using multiple logistic regression. Night-shift drivers were 1.84-fold more likely to develop MetS than night-shift security guards (p < 0.001).

**Table 4.** Odds ratio for prevalence of metabolic syndrome between security guards and drivers who underwent the Workers' Special Health Examination (WSHE) in South Korea, 2016

Variable	OR		95% CI	p
	security guards	drivers		
Prevalence of metabolic syndrome	1	1.844	1.722–1.974	<0.001

p – adjusted for age.

## DISCUSSION

In this study, the authors compared values related to health status and night-shift work duration between male drivers and security guards in the older worker population, based on the WSHE results from 2016. Drivers generally had longer duration of night-shift duty than security guards. Most security guards (85%) had worked for <5 years on night-shift duty. Drivers had unhealthy habits including smoking, binge drinking, and irregular exercise. Except for hypertension, the risk factors for developing CCVD, diabetes, hyperlipidemia, and obesity were more pronounced in drivers than in security guards. Approximately half of the drivers had a higher rate of MetS and a greater risk of CCVD than security guards. During the past decades, a growing body of evidence has suggested an association between shift work and several adverse health effects, including MetS [15] and overweight [16]. There was highly suggestive evidence that shift work increased the risk of myocardial infarction incidence [17] and A prior study found highly suggestive evidence in dose-response analysis and suggestive evidence in categorical analysis of the link between shift work and diabetes incidence [18].

Considering the working environment of professional night-shift drivers, their weight is expected to be higher than that of the general population and other occupational groups [19]. This is due to factors, such as prolonged sitting time, lack of physical activity, unhealthy eating habits, irregular working hours, poor sleep patterns, and chronic stress [20]. The duration of employment of security guards was relatively short compared to that of drivers in the authors' study; therefore, they would have been less affected by the impact of night-shift security work. Additionally, security positions in workplaces with a low likelihood of incidents require relatively few job skills, making them easily accessible to retirees. However, these positions may also be more vulnerable to turnover, and health status could be the primary factor for employment retention.

Compared with previous studies analyzing the general population, such as the Korea National Health and Nutrition Examination Survey (KNHANES), a nationwide cross-sectional survey conducted annually [21,22] revealed that drivers have a higher incidence of diabetes and obesity than security guards and the general older population (diabetes – 25.5%, obesity – 39.7%); however, the incidence of hypertension is lower in drivers than in the general population (55.9%). The incidence of binge drinking and hypercholesterolemia in the general population (binge drinking – 17.5%, hypercholesterolemia – 27.2%) was also higher than in drivers. Security guards appeared healthier than drivers and the general older population. However, if sex is not considered, male night-shift workers in their 60s in both occupations may be considered vulnerable. Kang et al. analyzed older population data from a nationwide health examination provided by the Korean National Health Insurance Services (NHIS) and found that the incidences of diabetes and hypertension in 2013 were 14.18% and 37.96%, respectively [10]. Both drivers and security guards in their 60s can be regarded as a vulnerable population compared to average NHIS subscribers in their 60s.

Occupations such as transportation, security, and nightshift work are associated with an increased risk of CCVD. International and domestic studies have been conducted regarding poor working environments and health risks. The risk of myocardial infarction in taxi drivers is higher than in other professional drivers [23]. Studies have re ported an elevated CCVD related risk, particularly for drivers. Long-term driving has been linked to elevated blood pressure, heart rate, and increased CCVD incidence [24,25]. A domestic study also showed that long-term driving is related to elevated blood pressure, heart rate, and catecholamine secretion, which can lead to an increased incidence of CCVD [26]. Similarly, typical security guard jobs for older males, often involving 12-hour or 24-hour shifts, are susceptible to CCVD risk [27].

In the authors' study, although drivers were generally less healthy than security guards, contrary to previous findings, neither group demonstrated a significantly higher CCVD risk than the general population. This may be because healthier workers are more likely to remain employed. Additionally, when individuals seek employment, healthy individuals are hired more frequently than those with less favorable health conditions [28]. Eom et al. [29] mentioned the healthy worker effect (HWE) as a confounding factor in a study that revealed that wage earners are healthier and have a lower incidence of CCVD than self-employed and unemployed workers, based on an analysis of Korean nationwide regular health examinations [29]. They stated that healthy workers would continue to work for a longer period and maintain a healthy status and that more opportunities are available for more medical services owing to the workers' health examination system in Korea. Additionally, unhealthy habits and chronic diseases can prompt early retirement, contributing to the HWE [30].

The authors' results show variations in the years of experience of drivers and security guards. Drivers and security guards in their 60s with <10 years of experience indicated that they were new employees recruited from another workplace where they had spent a long career. According to data from Statistics Korea, the number of self-employed men in their 60s in South Korea has roughly doubled over the past 20 years, surpassing 2 million in 2023 [31], largely because many retired older men have turned to jobs in the transportation sector, such as private taxis or delivery services [32]. This trend is due to advancements in car navigation technology and expansion of delivery brokerage systems, making these professions easily accessible to retirees, without requiring new qualifications or professional experiences beyond a driver license. Additionally, South Korea has a low crime rate relative to many other countries and employers tend to hire older individuals at lower wages for security work at facilities with low crime risks, reflecting the country's safe environment. As such, these jobs have relatively low entry barriers for those seeking new employment after retirement; however, these jobs require solo- or night-shift duties for longer periods. Driving at night or staying awake for extended periods may seem manageable for those entering the job, but if not properly managed, the accumulated strain can have serious consequences for one's health. Therefore, health status may represent an important criterion for entry into the labor force and for staying in the workplace. In particular, for security jobs, which have shorter employment durations, the fact that these positions have low entry barriers and do not require specialized skills actually indicates that they are difficult to maintain in the long term. As a result, workers in better health are more likely to succeed and remain employed longer.

Night-shift work can lead to various health risks, including cardiovascular and sleep disorders. This has influenced the inclusion of night-shift work as a specific hazardous factor that necessitates periodic health screening for workers in South Korea. Existing research surrounding night-shift work and the findings related to older male shift workers in the authors' study indicates that there are parallels in the risks associated with obstructive sleep apnea. Sleep apnea is more common in older adults and males, and numerous studies have shown that sleep apnea is a risk factor for CCVDs and related conditions [33]. Further research is required to investigate these associations, including their interactions and overlapping effects. In addition, when older male workers engage in night-shift work, they require careful attention.

The authors' study has the following limitations. The WSHE consist of WSHE results and final assessments based on hazardous factors and their target organs. Specific information, such as SES, is not included, and the data structure varies depending on the hazardous factors to which workers are exposed. Therefore, setting an appropriate population or control group is challenging. Additionally, there is no specific information about the night shift schedule. The health impacts of night-shift work vary depending on factors such as intensity, continuity, shift rotation, and day-night shift direction [34]. Moreover, there was no information on other variables in the WSHE data that could influence the study results, making it impossible to adjust for the effects of factors such as SES.

Therefore, the authors focuses on the most common job categories – driving and security positions – among men in their 60s who perform WSHE for night-shift work, excluding other characteristics related to shift work, and compared the risk factors for cardiovascular diseases between drivers and security guards. Additionally, the authors indirectly compared the results with data from KNHANES, a national sample survey, and analyses of health examination results conducted on a large scale by the NHIS. In addition, this study is limited by its cross-sectional nature, reliance on a single year of WSHE data, and the difficulty of accurately estimating the number of night-shift workers among the older male population. Further research using long-term data and considering cumulative occupational exposure effects is warranted to establish causal relationships and to better understand the health status of older night-shift workers.

Finally, this study used WSHE data from 2016, which might be considered somewhat dated. Since 2016, the labor participation rate of men in their 60s in South Korea has exceeded the Organization for Economic Co-operation and Development (OECD) average [35], the prevalence of obesity and abdominal obesity in Korean male in their 60s has increased [36], and the health issues of shift workers raised in this study remain relevant to the current context. Although, a major limitation is that the authors' results do not account for various changes in the employment market that occurred after 2016, such as the expansion of remote work following the COVID-19 pandemic and the increase in unmanned transportation due to technological advancements.

Despite these limitations, the authors' results are noteworthy. This study used nationwide WSHE data to analyze CCVD-related factors in drivers and security guards, who account for most of the older male population working nightshifts, as WSHE practitioners. Currently, Korean workers have longer working hours than workers in other developed countries, and various occupations require nightshifts [9]. Furthermore, Korea has a notably low unemployment rate among the older worker labor force [35]. With a rapidly changing population structure due to aging, the demand for an older employed population is expected to continue. Therefore, healthcare for night-shift workers in older populations is an important public health issue. Rather than relying solely on consistent national health screening and result notifications, proactive management strategies tailored to CCVD risk levels of cardiovascular and cerebrovascular diseases are required. Additionally, further epidemiological studies are needed to assess the health impacts of specific occupations while excluding the HWE.

## CONCLUSIONS

Driving and security jobs make up the largest proportion of 60-year-old workers who have participated in WSHE related to shift work in South Korea. They work alone during night shifts and coping with medical emergencies may be difficult in the workplace. Thus, understanding and managing the health status of these workers is important because health risks can have damaging effects and lead to fatal accidents that can affect public safety. This study showed that the risk of developing CCVD is higher among drivers than among security guards. Moreover, the authors found that the incidences of obesity and diabetes were higher among drivers than among the general population. However, verifying the occupational hazards due to the HWE and certain other limitations of this study were difficult. Further research should be conducted to accurately understand the effects of the HWE and health risks to older drivers and security guards working nightshifts.
